# An *SCN1A* gene missense variant in a Chinese Tujia ethnic family with genetic epilepsy with febrile seizures plus

**DOI:** 10.3389/fneur.2023.1229569

**Published:** 2023-07-27

**Authors:** Ling Li, Lamei Yuan, Wen Zheng, Yan Yang, Xiong Deng, Zhi Song, Hao Deng

**Affiliations:** ^1^Health Management Center, The Third Xiangya Hospital, Central South University, Changsha, China; ^2^Center for Experimental Medicine, The Third Xiangya Hospital, Central South University, Changsha, China; ^3^Department of Neurology, The Third Xiangya Hospital, Central South University, Changsha, China; ^4^Disease Genome Research Center, Central South University, Changsha, China

**Keywords:** genetic epilepsy with febrile seizures plus, *SCN1A*, whole exome sequencing, missense variant, genetic diagnoses

## Abstract

Genetic epilepsy with febrile seizures plus (GEFSP) is a familial epileptic syndrome that is genetically heterogeneous and inherited in an autosomal dominant form in most cases. To date, at least seven genes have been reported to associate with GEFSP. This study aimed to identify the disease-causing variant in a Chinese Tujia ethnic family with GEFSP by using whole exome sequencing, Sanger sequencing, and *in silico* prediction. A heterozygous missense variant c.5725A>G (p.T1909A) was identified in the sodium voltage-gated channel alpha subunit 1 gene (*SCN1A*) coding region. The variant co-segregated with the GEFSP phenotype in this family, and it was predicted as disease-causing by multiple *in silico* programs, which was proposed as the genetic cause of GEFSP, further genetically diagnosed as GEFSP2. These findings expand the genetic and phenotypic spectrum of GEFSP and should contribute to genetic diagnoses, personalized therapies, and prognoses.

## 1. Introduction

Genetic epilepsy with febrile seizures plus (GEFSP, also called GEFS+), previously known as generalized epilepsy with febrile seizures plus, was first defined by Scheffer and Berkovic as an inherited epileptic syndrome with genetic and phenotypic heterogeneity (1, 2). It is a familial epilepsy syndrome clinically characterized by various types of seizures, including fever-associated and afebrile seizures (3, 4). GEFSP includes a wide range of subtypes with varying prevalence, of which the estimated prevalence of febrile seizures (FS) is 3–4%, and the overall prevalence is unclear (5, 6). The most common phenotype in the GEFSP pedigrees is FS, which occurs between the age of 3 months and 6 years, followed by febrile seizures plus (FS+), in which episodes with fever persist beyond 6 years or afebrile seizures occur (7). The diagnosis is based on the occurrence of two or more family members that show phenotypes in the spectrum, including FS, FS+, FS/FS+ with various seizures (such as absence, atonic, myoclonic, and partial seizures), and myoclonic-atonic epilepsy, along with Dravet syndrome (8–10). The inheritance patterns include autosomal dominant, autosomal recessive, and complex inheritance in which several genes are involved, accompanied by possibly environmental contributions. In pedigrees with monogenic variants, most of them follow autosomal dominant patterns, and the minority abides by autosomal recessive traits. In some small families, the most common pattern may be complex inheritance (11, 12). To date, at least 11 genetic loci are recorded for GEFSP in the Online Mendelian Inheritance in Man (OMIM, https://www.omim.org/). Variants in the critical regions of at least seven genes, including the sodium voltage-gated channel beta subunit 1 gene (*SCN1B*), the sodium voltage-gated channel alpha subunit 1 gene (*SCN1A*), the gamma-aminobutyric acid type A receptor subunit gamma2 gene (*GABRG2*), the gamma-aminobutyric acid type A receptor subunit delta gene (*GABRD*), the syntaxin 1B gene (*STX1B*), the hyperpolarization-activated cyclic nucleotide-gated potassium and sodium channel 1 gene (*HCN1*), and the hyperpolarization-activated cyclic nucleotide-gated potassium and sodium channel 2 gene (*HCN2*), were reported to be responsible for GEFSP (13–19). Of these, the *SCN1A* gene is the most clinically relevant and most frequently reported disease-causing gene for the GEFSP spectrum, and ~11% of reported pedigrees were caused by its variants (20).

In this study, we identified a heterozygous missense variant c.5725A>G (p.T1909A) in the *SCN1A* gene as the causative variant in a Chinese Tujia ethnic family with GEFSP.

## 2. Materials and methods

### 2.1. Participators and clinical evaluations

A non-consanguineous Chinese Tujia ethnic family located in the west of Hunan province was recruited from the Third Xiangya Hospital, Central South University (Changsha, China). The GEFSP diagnosis was based on clinical features, family history, electroencephalography (EEG), and genetic testing. The medical history of the patients was collected. Routine physical examinations and EEG were performed on the proband. All individuals had signed the written informed consent before the peripheral venous blood samples were acquired. The approval of this study was received from the Institutional Review Board of the Third Xiangya Hospital, Central South University, Changsha, China.

### 2.2. DNA extraction and exome capture

**Figure 1:**
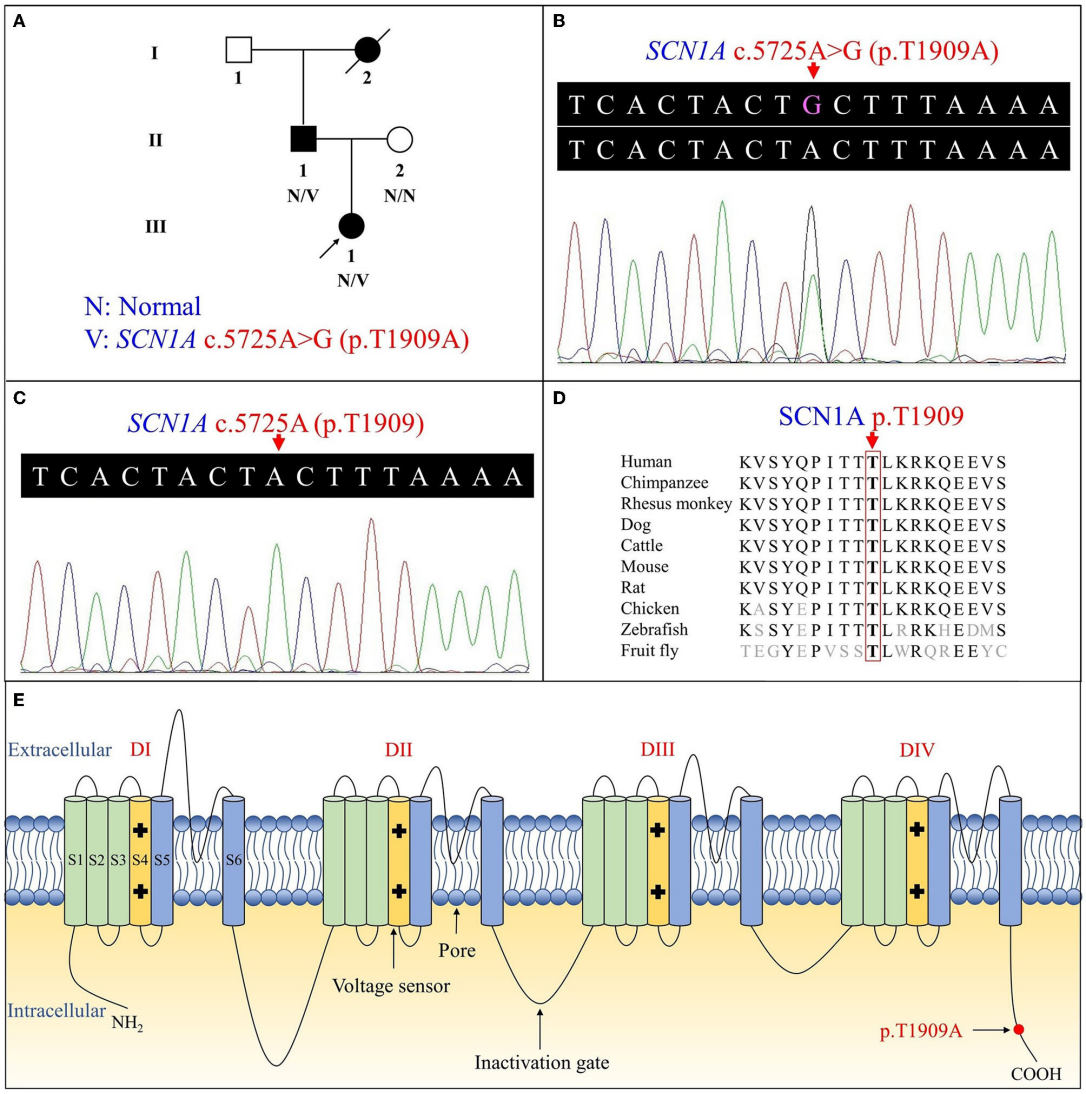
Pedigree figure of the Chinese Tujia ethnic family with genetic epilepsy with febrile seizures plus (GEFSP) and the variant analysis. **(A)** A pedigree figure of the family. **(B)** *SCN1A* sequence of the proband (III:1) with heterozygous c.5725A>G (p.T1909A) variant. **(C)** Normal *SCN1A* sequence of the unaffected member (II:2). **(D)** SCN1A p.T1909 amino acid residue is conserved in multiple species. **(E)** Schematic structure of SCN1A protein and the location of the p.T1909A variant. S1–S6 represent six alpha-helical transmembrane segments, and DI–DIV represent four homologous domains.

The genomic DNA (gDNA) was extracted from peripheral venous blood samples by using the standard phenol-chloroform extraction method as previously described (21). The gDNA samples of II:1 and III:1 (Figure 1A) were randomly fragmented to 150 bp-250 bp using Covaris technology and prepared for whole exome sequencing (WES). The ends of DNA fragments were repaired, and the “A” base was ligated at the 3′-end of each strand. Adapters were added to both ends of the end-repaired DNA for PCR amplification and further sequencing. The products were then purified and hybridized to the exome array. Hybridized fragments were used for circularization, and non-specific fragments were cleaned out (22). DNA nanoballs were produced by rolling circle amplification. The qualified captured library was loaded on the BGISEQ-500 sequencing platform, and sequencing-derived raw image files were analyzed by BGISEQ-500 base-calling software, performed by the BGI-Shenzhen (Shenzhen, China).

### 2.3. Read mapping and variant analysis

The clean data were produced by noise-decrease data filtering on raw data and then aligned to the human reference genome (GRCh37/hg19) by Burrows–Wheeler Aligner (BWA, v0.7.15) (23). Picard tools (v2.5.0) were used to label and remove duplicate reads. The Genome Analysis Toolkit (GATK, v3.3.0) was used for local realignment and base quality score recalibration (24, 25). Single nucleotide polymorphisms (SNPs) and insertions-deletions (indels) were called using HaplotypeCaller of GATK and then annotated with the SnpEff tool (26). All candidate variants were screened and analyzed by the public databases, including the Single Nucleotide Polymorphism database (dbSNP, build 141), the 1000 Genomes Project, the National Heart, Lung, and Blood Institute Exome Sequencing Project 6500 (NHLBI ESP6500), the Exome Aggregation Consortium (ExAC), the Genome Aggregation Database (gnomAD), the China Metabolic Analytics Project (ChinaMAP), Human Gene Mutation Database (HGMD), and ClinVar, along with the in-house BGI exome database including 1,943 controls (27–33). The MutationTaster (http://www.mutationtaster.org/), Protein Variation Effect Analyzer (PROVEAN, http://provean.jcvi.org/), Sorting Intolerant from Tolerant (SIFT, http://provean.jcvi.org/), Polymorphism Phenotyping v2 (PolyPhen-2, http://genetics.bwh.harvard.edu/pph2/), and MutationAssessor (http://mutationassessor.org/r3/) were used to obtain the predicted pathogenic effects of variants (34–37). Sanger sequencing was employed to validate the causative variant with an ABI3500 sequencer (Applied Biosystems, Foster City, CA, USA). Primers used for PCR amplification and sequencing were designed and analyzed by the Primer3 program and Primer-BLAST, and the primer sequences for detecting the disease-associated variant are as follows: forward, 5′-GTGACCGGATCCACTGTCTT-3′ and reverse, 5′-GCTTTAAAAGGTGGCGTCTG-3′. The NCBI Basic Local Alignment Search Tool (BLAST, https://blast.ncbi.nlm.nih.gov/Blast.cgi) was used to conduct conservation analysis among multiple species (38). Wild-type and mutant protein structures were predicted by SWISS-MODEL (https://swissmodel.expasy.org/) and displayed by PyMOL software (v2.5.2, Schrödinger, LLC, New York, NY, USA) (39). The American College of Medical Genetics and Genomics (ACMG) guidelines for the sequence variant interpretation were utilized to classify the identified variant (40).

## 3. Results

### 3.1. Clinical findings

The proband (III:1) was a 22-year-old female with unremarkable spontaneous vaginal delivery and development. Her first seizure occurred at the age of 2 years when she suffered from an upper respiratory tract infection and had a fever of ~38°C. After that, she had recurrent episodes when she caught a fever or felt nervous. She has suffered from attacks of afebrile seizures (AFS) since the age of 14 years. The episodes can manifest as moderate generalized tonic-clonic seizures, with a mean duration of 2–7 min, and focal impaired awareness seizures, with a mean duration of 1–1.5 min. The ambulatory EEG showed interictal epileptiform discharges: bilateral slow waves, sharp waves, and sharp-and-slow wave complexes (41). Irregularly antiepileptic drug lamotrigine treatment not adhering to medical advice was applied, and the efficacy was not good. The self-reported medical history of her father (II:1) revealed fever-associated seizures at 7 months and spontaneous remission before the age of 14 years. Focal impaired awareness seizures began at the age of 7 years, and no AFS were claimed. Detailed features of patients in this family are presented in Table 1.

**Table 1:** Clinical features and genotypes of patients.

**Case**	**II:1**	**III:1**
Sex	Male	Female
Age (years)	49	22
Ethnic background	Chinese Tujia ethnic	Chinese Tujia ethnic
Variant	c.5725A>G (p.T1909A)	c.5725A>G (p.T1909A)
Zygosity	Heterozygote	Heterozygote
Inheritance	Maternal?	Paternal
Phenotype	FS with focal impaired awareness seizures	FS+ with focal impaired awareness seizures
FS onset/remission	7 months/14 years	2 years/no
FS episode count	3	30
AFS onset/remission	No/not applicable	14 years/no
AFS episode count	0	20
Seizure type	GTCS and FIAS	GTCS and FIAS
Seizure duration	2–7 min (FS) and 0.5–1 min (FIAS)	2–7 min (GTCS) and 1–1.5 min (FIAS)
Seizure frequency	Once per 2–3 years	Once per month (GTCS) and twice per 3 months (FIAS)
Heat sensitivity (fever-association)	100%	Before age 14: 100%, after age 14: < 50%
Familial history	Yes	Yes
Pregnancy and delivery	Possible malnutrition in pregnancy (self-reported)	Normal
Psychomotor development	Normal	Normal
Neurological examination	Not available	Normal
Electroencephalogram	Not available	Interictal epileptiform discharges
Brain imaging	Not available	Normal MRI
Antiepileptic drug response	Not applicable (remission without antiepileptic drugs)	Slightly shortened duration and decreased frequency on lamotrigine

FS, febrile seizures; AFS, afebrile seizures; FS+, febrile seizures plus; GTCS, generalized tonic-clonic seizures; FIAS, focal impaired awareness seizures; MRI, magnetic resonance imaging.

### 3.2. Genetic analysis

**Figure 2:**
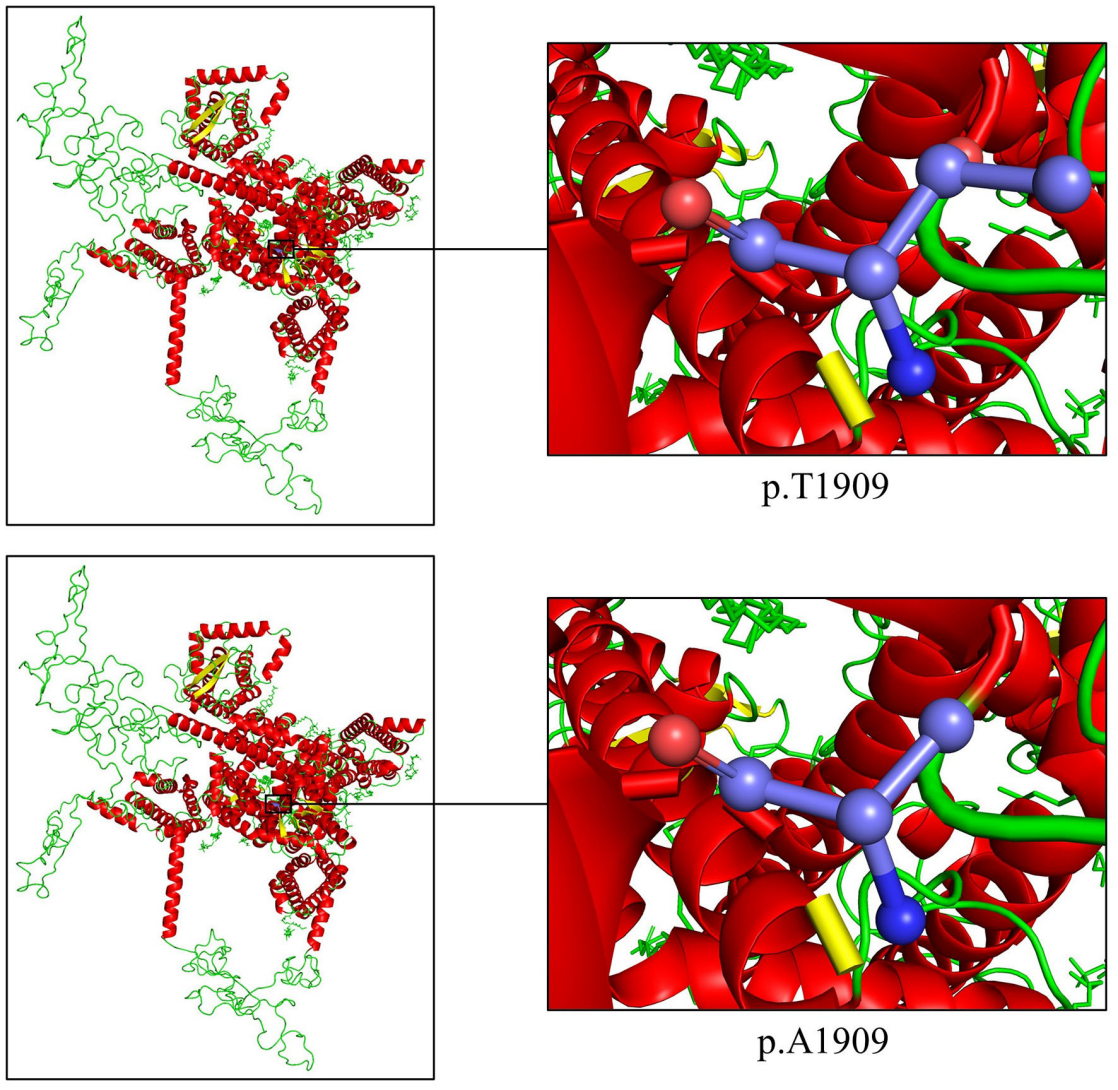
Cartoon model of the SCN1A protein structure by PyMOL 2.5.2 based on the SWISS-MODEL: the threonine and the altered alanine at position 1909 are shown as ball-and-stick models.

Overall, the WES of the two patients generated an average of 203.59 million effective reads. Of these, ~99.95% were mapped to the human reference genome. The target sequence covered 99.68% of bases at ≥10×, and the average sequencing depth was 256.39×. A total of 98,432 SNPs and 17,160 indels were identified in the father (II:1), and ~106,543 SNPs and 18,860 indels were detected in the proband (III:1). A variant filtering scheme, described in recent studies, was applied for detecting the potential disease-associated variant for patients. Common variants registered in dbSNP, 1000 Genomes Project, and NHLBI ESP6500 with an allele frequency of >0.5% were excluded, and damaging variants predicted by *in silico* tools were reserved. Using the mentioned criteria, only a heterozygous variant of the *SCN1A* gene (NM_001353948.2), c.5725A>G (p.T1909A), shared by two patients, was judged as the most likely disease-causing variant and predicted to be deleterious by bioinformatics (Table 2). Sanger sequencing corroborated the variant in the patients (II:1 and III:1, Figure 1B) and revealed the absence of the variant in the proband's unaffected mother (II:2, Figure 1C). Conservation analysis showed the high conservation of the mutated p.T1909 residue among 10 species (Figure 1D), and the conformation change caused by the variant was shown in Figure 2. Based on the above evidence, the variant c.5725A>G (p.T1909A) in the *SCN1A* gene appears to be accountable for the GEFSP in this family. According to ACMG guidelines, the *SCN1A* gene variant c.5725A>G (p.T1909A) was classified as a “likely pathogenic” (PS1 + PM2 + PP1 + PP3) variant.

**Table 2:** Identification of the disease-associated variant in the patients.

**Item**	**Information**
Gene	*SCN1A*
Exon	27
Nucleotide change	c.5725A>G
Amino acid change	p.T1909A
Zygosity	Heterozygote
Variant type	Missense variant
dbSNP141	Absence
1000G	Absence
NHLBI ESP6500	Absence
ExAC	Absence
gnomAD	Absence
ChinaMAP	Absence
ClinVar	Absence
HGMD	CM173503
In-house BGI exome database	Absence
MutationTaster (probability value, prediction)	0.999, disease causing
PROVEAN (score, prediction)	−4.60, deleterious
SIFT (score, prediction)	0.000, damaging
PolyPhen-2 (score, prediction)	0.999, probably damaging
MutationAssessor (FI score, prediction)	4.03, high function impact
Classification (ACMG guidelines)	Likely pathogenic

*SCN1A*, the sodium voltage-gated channel alpha subunit 1 gene; dbSNP141, Single Nucleotide Polymorphism database build 141; 1000G, 1000 Genomes Project; NHLBI ESP6500, National Heart, Lung, and Blood Institute Exome Sequencing Project 6500; ExAC, Exome Aggregation Consortium; gnomAD, Genome Aggregation Database; ChinaMAP, China Metabolic Analytics Project; HGMD, Human Gene Mutation Database; PROVEAN, Protein Variation Effect Analyzer; SIFT, Sorting Intolerant from Tolerant; PolyPhen-2, Polymorphism Phenotyping v2; FI, functional impact; ACMG, American College of Medical Genetics and Genomics.

## 4. Discussion

GEFSP is a familial epileptic syndrome with remarkable genetic and phenotypic heterogeneity (42). It was first described in a large Australian family having an unusual concentration of generalized epilepsy and FS (43). Since the *SCN1B* gene was reported as the first causative gene of GEFSP in 1998, a large number of GEFSP-related variants have been identified in seven pathogenic genes, in which the *SCN1A* gene is the most frequently reported and most clinically relevant one (44).

In this study, we identified the *SCN1A* missense variant c.5725A>G (p.T1909A) in two members of the Chinese Tujia ethnic family via WES and Sanger sequencing, which was found to co-segregate with the phenotype in the family. The proband had AFS and uncontrolled seizures in adulthood, and her father had self-limited seizures, in favor of the phenotypic heterogeneity of GEFSP, genetically diagnosed as GEFSP2.

The *SCN1A* gene, located on chromosome 2q24.3, includes 27 exons, encoding the protein sodium voltage-gated channel alpha subunit 1 (Na_v_1.1) with 2,009 amino acids (~229 kDa) (45). The Na_v_1.1 includes four homologous domains (I-IV), each containing six alpha-helical transmembrane segments (S1-S6), in which the S4 is a voltage sensor, and the S5 and S6 form the ion-selective pores (Figure 1E) (46). It is highly expressed in the brain, especially the prefrontal cortex, and forms the voltage-dependent sodium channel with beta subunits as one of the primary sodium channels in the central nervous system (http://biogps.org/), which plays a crucial role in the initiation and propagation of action potentials in neurons (47, 48).

To date, more than 2,584 variants in the *SCN1A* gene have been recorded, and most are deemed responsible for GEFSP phenotypes (HGMD, http://www.hgmd.cf.ac.uk/ac/index.php). The c.5725A>G variant was located at the exon 27 of the *SCN1A* gene. It was absent in public databases including dbSNP, the 1000 Genomes Project, NHLBI ESP6500, ExAC, gnomAD, ChinaMAP, ClinVar, and the in-house BGI exome database. The variant was predicted as deleterious or probably damaging by multiple *in silico* programs including MutationTaster, PROVEAN, SIFT, PolyPhen-2, and MutationAssessor. The threonine at position 1,909 was phylogenetically conserved among varied species from fruit flies to human beings. The polar hydrophilic to non-polar hydrophobic residue change may affect the tertiary structure and impact normal function (49). The p.T1909A variant is located in the cytoplasmic region of the Na_v_1.1 C-terminal domain, which contains binding sites of interaction proteins and plays an important role in channel inactivation (50).

A *de novo* c.5725A>G (p.T1909A) variant was reported in a patient with focal epilepsy, recorded in HGMD (CM173503) (44). Moreover, a missense variant involving the same residue, c.5726C>T (p.T1909I, rs121918793), was reported in a 7-month-onset female with severe myoclonic epilepsy of infancy (SMEI, i.e., Dravet syndrome) (51).

Functional studies indicated that different *SCN1A* variants alter sodium channel properties and functions in different ways and cause distinctive effects on the sodium channel activity, thus affecting the selection of antiepileptic drugs (52). Both gain-of-function (GOF) and loss-of-function (LOF) effects could be responsible for *SCN1A*-associated epilepsies. In human tsA201 cells with p.R1648C or p.F1661S variant, a small non-inactivating persistent inward current during depolarization was reported to lead to neuron hyperexcitability, which exhibited a GOF effect in the sodium channels (53). GOF-related epilepsy can usually be relieved by commonly prescribed antiepileptic drugs that inhibit sodium channels, such as carbamazepine and phenytoin (54). *SCN1A* LOF variants mainly impair bipolar GABAergic inhibitory interneurons and lead to diminished inhibition (55). The application of GABA transaminase inhibitors such as valproic acid or GABA receptor-positive allosteric modulators, such as pentobarbital, appears to be effective, while the sodium channel blocker may provoke seizure aggravation (56). In human tsA201 cells, the SMEI-associated p.T1909I variant exhibited a mixture mechanism of GOF and LOF, with increased persistent current and reduction of current density (57). In this study, disease symptom remission and seizure frequency reduction after lamotrigine application in the proband were unsatisfactory. Due to the same biophysical property change (polar hydrophilic to non-polar hydrophobic) of p.T1909I and p.T1909A variants, the same mechanism may be shared. Limited response to medication may be due to the use of a single sodium channel blocking antiepileptic drug. The prescription of GABA transaminase inhibitors or GABA receptor-positive allosteric modulators might be beneficial for the sufferers.

Genetic deficient animal models verified the important role of the *SCN1A* gene in epilepsy development. The *drosophila SCN1A* p.K1270T variant knock-in model showed that when the temperature rose, the deactivation threshold for persistent sodium currents reversibly shifted to a more negative voltage, causing sustained depolarizations in GABAergic inhibitory interneurons and leading to reduced inhibitory activity in the brain (58). Heterozygous (*Scn*1*a*^+/−^) mice had spontaneous seizures and occasional deaths beginning on postnatal day 21, attributed to haploinsufficiency of Na_v_1.1 channels, and homozygous *Scn1a* knockout (*Scn*1*a*^−/−^) mice developed severe ataxia and seizures and died on postnatal day 15, corresponding to the LOF effect (59). In a bacterial artificial chromosome transgenic *SCN1A* p.R1648H variant mouse model, experiments showed a delayed recovery of channel inactivation only in inhibitory neurons, suggesting the cell type-dependence of *SCN1A* mutation and the p.R1648H variant leading to a reduction in inhibitory neurons excitability (60).

## 5. Conclusion

In summary, a missense variant c.5725A>G (p.T1909A) in the *SCN1A* gene was identified in a Chinese Tujia ethnic family with GEFSP, further classified as GEFSP2. The different phenotypes of the same variant in the family show the heterogeneity of GEFSP. These findings confirmed the *SCN1A*-associated GEFSP, which may expand the genetic and phenotypic spectrum of GEFSP and improve genetic diagnoses, personalized therapies, and prognoses. The combination of WES and Sanger sequencing efficiently provided a timely diagnosis and indicated management for those with clinically and/or genetically suspected GEFSP. Additional *in vitro* studies and the establishment of genetically deficient animal models to explore the functional effect of human *SCN1A* variants may help to further illuminate potential pathogenic mechanisms.

## Data availability statement

Data of this project can be accessed after an approval application to the China National Genebank (CNGB, https://db.cngb.org/cnsa/). Please refer to https://db.cngb.org/, or email: CNGBdb@cngb.org for detailed application guidance. The accession code CNP0004397 should be included in the application.

## Ethics statement

The studies involving human participants were reviewed and approved by the Institutional Review Board of the Third Xiangya Hospital, Central South University, Changsha, China. Written informed consent to participate in this study was provided by the participants' legal guardian/next of kin. Written informed consent was obtained from the individual(s), and minor(s)' legal guardian/next of kin, for the publication of any potentially identifiable images or data included in this article.

## Author contributions

LL, LY, and HD conceived and designed this study and wrote the manuscript. WZ, YY, and ZS collected the patient samples and clinical data. LL, LY, WZ, and XD performed the experiments. LL, LY, WZ, XD, and HD analyzed the data. The final version of the manuscript was read and approved by all authors.
